# Health Professionals' and Postpartum Women's Perspectives on Digital Health Interventions for Lifestyle Management in the Postpartum Period: A Systematic Review of Qualitative Studies

**DOI:** 10.3389/fendo.2019.00767

**Published:** 2019-11-08

**Authors:** Siew Lim, Andrea Tan, Seonad Madden, Briony Hill

**Affiliations:** ^1^Monash Centre for Health Research and Implementation, School of Public Health and Preventive Medicine, Monash University, Clayton, VIC, Australia; ^2^School of Health Sciences, College of Health and Medicine, University of Tasmania, Newnham, TAS, Australia

**Keywords:** digital health interventions, eHealth, postpartum women, weight, lifestyle management, qualitative, systematic review

## Abstract

**Objective:** To explore postpartum women and health professionals' perspectives of digital health interventions (DHIs) for lifestyle management in postpartum women.

**Design:** A systematic review and thematic synthesis of peer-reviewed qualitative studies. Relevant databases were searched from 1990 to 2019. Study quality was appraised using the Critical Appraisal Skills Programme (CASP) Qualitative Checklist.

**Setting and participants:** Studies describing postpartum women's or health professionals' views regarding DHIs for lifestyle management in postpartum women.

**Findings:** Nine studies with postpartum women were included in the thematic synthesis. Four common themes emerged: “personal facilitators and barriers to lifestyle modification,” “intervention-related strategies for lifestyle modification,” “user experience of the technology,” “suggestions for improvement.” The review indicated that DHIs are highly acceptable among postpartum women. Postpartum women valued behavior change strategies that were delivered through DHIs including goal-setting and self-monitoring, however personal barriers such as lack of motivation or childcare priorities were cited.

**Key conclusions and implications for practice:** DHIs should be considered for lifestyle management in postpartum women. The development of DHIs should focus on delivering behavior change strategies and addressing practical barriers faced by postpartum women.

## Introduction

Individuals with overweight or obesity now represent over half of the global adult population (1). The reproductive life phase is recognized as a key driver of weight gain in women (2), with about half of all women of reproductive age entering pregnancy above optimal weight status (3, 4). Furthermore, up to 50% of women gain excessive weight during pregnancy, predisposing them to postpartum weight retention [PPWR; (3)], with up to half of women 4.5 kg or more heavier than their pre-pregnancy weight by one year postpartum (5). Postpartum weight retention, in turn, leads to high preconception weight status entering subsequent pregnancies (2). High preconception weight status, excessive gestational weight gain, and PPWR are associated with a host of adverse maternal outcomes including infertility (6), pregnancy complications such as gestational diabetes and hypertensive disorders of pregnancy (7, 8), and cesarean delivery (9), as well as poorer offspring outcomes including stillbirth (10), and macrosomia (7). Furthermore, there are long-term consequences for the development of overweight or obesity in offspring (11). Consequently, the postpartum period is an opportune time to intervene to promote the return to pre-pregnancy weight and potentially stem the intergenerational cycle of obesity.

The postpartum period, beginning immediately at birth, represents a period of significant physical and emotional change, with tremendous responsibilities, challenges, and expectations (12–14). Although some may assume that breastfeeding would facilitate weight loss after birth (15), in reality, competing demands mean that prioritizing the return to a healthy body mass index (BMI) can be difficult during this period (13). Other barriers to lifestyle or weight management cited by postpartum women include lack of personal effort, tight finances, low self-esteem, and lack of social support (16). Digital health interventions (DHIs) may be a potential solution to overcome some of the barriers faced by women in the postpartum period that prevent healthy lifestyle behaviors that facilitate weight management (17). DHIs describe health interventions that incorporate the use of information and communications technologies (ICT), which include Mobile Health and Electronic Health (eHealth) interventions (18). DHIs are also appealing to service providers as they may alleviate resource strains on the healthcare system and costs associated with in-person delivery (19). A 2017 meta-analysis of the efficacy of eHealth postpartum weight loss interventions indicated that DHIs resulted in 2.55 kg greater weight loss than controls (19).

DHIs may include delivery modes such as websites, phone calls, text messages, and electronic devices (e.g., phones or tablets); these have become more common for postpartum women in recent times (19). Indeed, 99% of postpartum women own a mobile phone and up to 86% of women have access to the Internet via smartphone or Internet connection in their household (20, 21). Favored attributes of DHIs include their convenience and ease of use (22). However, some research suggests that engagement in DHIs by postpartum women remains sub-optimal, impacting intervention effectiveness (22, 23). In a clinical trial, women who received an eHealth intervention in the form of an application (app) were not able to significantly decrease PPWR compared to participants receiving standard care (23). However, in this study, women with high intervention adherence achieved significant reduction in postpartum weight compared to their control counterparts, suggesting that improved adherence could improve weight management. One barrier to the engagement of individuals with DHIs has been attributed to a poor fit between the digital product and users' needs (24).

Consequently, there is a need to understand and explore how to increase engagement and adherence with postpartum DHIs. Importantly, the perspectives and needs of postpartum women themselves are essential to understanding how to optimize delivery of DHIs for this population. Furthermore, a recent meta-analysis of intervention components within interventions to reduce PPWR highlighted that the presence of a health professional was a key factor in intervention success (25). Hence, DHIs with health professional input may be particularly effective. The perspectives of health professionals will be invaluable in designing DHIs for lifestyle management in the postpartum period. Indeed, it is increasingly recognized that understanding stakeholders' views are highly important when designing, implementing and evaluating interventions (26). To date, the perspectives of both women and health professionals in the context of DHIs for postpartum weight loss have not been comprehensively described. Thus, the aim of this study was to conduct a systematic review to explore the perspectives of postpartum women and health professionals on DHIs for lifestyle management in postpartum women.

## MethodS

### Information Sources and Search Strategy

The systematic review was conducted in accordance with the Preferred Reporting Items for Systematic Reviews and Meta-Analyses (PRISMA) guidelines (27) and was registered on PROSPERO (registration number CRD42019129134). The search strategy was developed in consultation with a university librarian and the search was conducted in Medline Complete, PsycINFO, CINAHL Complete, and Embase. The search strategy combined the concepts of postpartum period (including postnatal, post-pregnancy, and following childbirth or pregnancy), DHIs (including mobile health, electronic health, telephone, or other digital intervention), study design (qualitative, interview, or focus groups), and weight (including weight retention or loss, BMI, overweight, obesity, diet, nutrition, or physical activity), see Box 1. The full search strategy for the Medline database is presented in Appendix 1. The search was conducted in February 2019 and limited to 1990–2019, however no language restrictions were applied. The rapid rise and acceptance of technological innovations after 1990 was considered rationale for this date restriction (19).

Box 1 **Search strategy.**

Concept 1—postpartum period

Postpartum period OR postpartum OR post-partum OR postnatal OR post-natal OR puerperium OR postpartal OR post-partal OR lactating OR lactation OR “nursing women” OR breastfeeding OR breast-feeding OR “after birth” OR “following pregnancy OR postpregnancy OR post pregnancy OR “following childbirth” OR “after delivery” OR “post childbirth”

**AND**

Concept 2—digital health intervention

m-health OR M-health OR E-health OR ehealth OR ICT OR mobile OR web* OR telephone OR phone* OR digital

**AND**

Concept 3—study design

Qualitative OR survey* OR interview* OR focus group*

**AND**

Concept 4—weight/lifestyle

Weight OR “weight retention” OR “weight loss” OR BMI OR “body mass index” OR overweight OR obes* OR “body fat” OR adiposity OR “waist circumference” OR dietary OR diet OR nutrition OR “healthy eating” OR “physical* active*” OR exercise*

### Inclusion and Exclusion Criteria

Studies were eligible if they included the perspectives of postpartum women or health professionals including obstetricians, midwives, general practitioners, dietitians, and physiotherapists. Any form of qualitative study such as open-ended surveys, interviews, or focus groups were included. Studies were required to report on the opinions, attitudes, perspectives or experiences of the participants, specifically about lifestyle, diet, physical activity, and/or other weight-related interventions that were delivered electronically, which, for the purposes of this review, included websites, phone calls, text messages, videos, social media, and personal device applications.

Studies were excluded if the focus was not on postpartum period (e.g., pregnancy with no postpartum perspective), if solely quantitative data were collected, or where the number of face-to-face or non-electronically delivered consultations exceeded the number of sessions delivered electronically. Editorials, narrative reviews, conference abstracts, letters, and commentaries were also excluded.

### Study Selection and Screening

After removal of duplicates, titles, and abstracts were screened in duplicate using Covidence systematic review software (Veritas Health Innovation, Melbourne, Australia, available at www.covidence.org) by two authors (AT and SM). Remaining full text papers were read in full and screened by two authors (AT and SM). In both cases, a third author (SL) was consulted when consensus could not be reached. At the full text screening stage, reasons for exclusion were noted.

### Quality Assessment

Quality assessment of included studies was evaluated using the Critical Appraisal Skills Program (CASP) Qualitative Checklist (28) by one author (AT), with a 10% sub-sample completed by a second author (SL) to establish reliability; 100% agreement was achieved between the two authors. The CASP checklist was developed through consultation with experts and piloted in the format it would be used. The CASP checklist allows critique of validity, results, and clinical relevance; a recent evaluation supported the use of this format (28). Studies were evaluated as met/not met/unsure, across the following criteria: clear aims, qualitative methodology, design, recruitment, data collection, pre-existing relationship, ethical consideration, rigor of data analysis, findings, and value (contribution) of the research.

### Data Extraction and Synthesis of Results

Data were extracted from the reviews into a piloted form by one author (AT), with a 10% sub-sample completed by a second author (BH) to establish reliability; 84% agreement was achieved between the two authors, with discussion to resolve disagreements. The following information was extracted: author, year of publication, country, setting, sample size, participant details (sampling frame, age, BMI, postpartum stage, inclusion, and exclusion criteria, withdrawals/loss to follow-up, and medical history), and key findings.

Thematic synthesis was conducted in a manner consistent with other qualitative systematic reviews (29, 30). Descriptive codes and analytical themes and subthemes were identified inductively with open coding. Codes, sub-themes, and themes were processed iteratively using spreadsheets, mind-mapping, and note-taking until defined themes were apparent, and any discrepancies were resolved. Themes were then grouped into categories. Two researchers (SL and BH) conducted the analyses independently and then collaboratively until consensus on the key themes and categories was achieved.

## Results

### Study Selection

**Figure 1:**
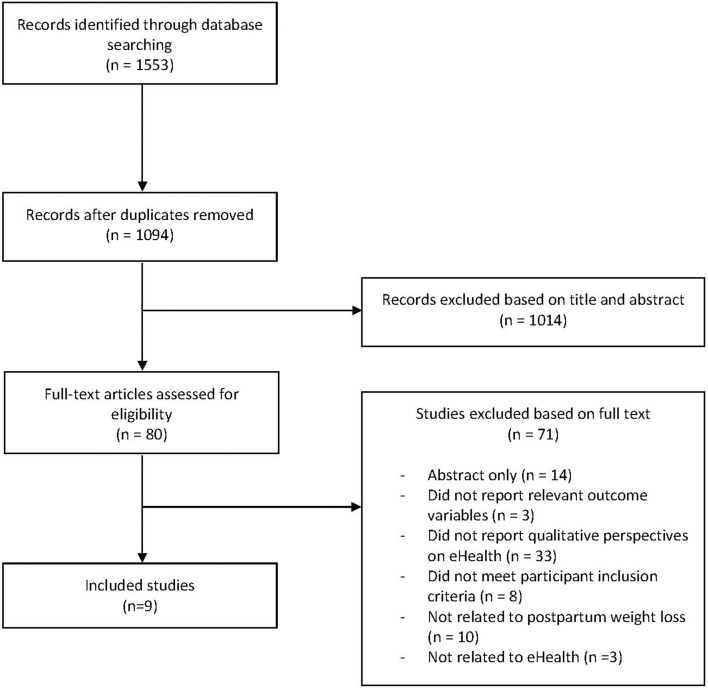
Flow chart of study selection.

The search identified 1,553 records. After removing duplicates and on the basis of title and abstract alone, 80 full texts were evaluated for inclusion and 9 studies were included in this review (Figure 1). Reasons for exclusions are shown in Appendix 2.

### Study Characteristics

Characteristics of included studies are summarized in Table 1 and Appendix 3. All included studies reported on the perspectives of postpartum women. There were no studies involving health professionals. Of the included studies, four were conducted in Australia (31–33, 37), three in the USA (34–36), one in England (22), and one in Bangladesh (38). Five studies conducted interviews (22, 31, 32, 36, 37), two conducted focus groups (33, 35), one conducted a survey (34), and one conducted a combination of surveys, focus groups, and interviews (38). In all studies, participants were within the first year after birth. Delivery modes of the DHIs in these studies included websites (22, 31, 34, 36), social media (Facebook) (31), smartphone applications (31, 33, 35), telephone counseling (31, 32, 38), group blog (31), text message (34, 36), voice message (38), video (22, 34, 37), and email (34, 36). Most studies utilized more than one delivery mode in the DHIs. The median response rate of the included studies was 45%.

**Table 1:** Characteristics of the studies included in the qualitative synthesis.

**References**	**Country**	**Participants characteristics**	**Digital health intervention platforms**	**Data collection**	**Sample size**	**Response rate (%)**
van der Pligt et al. (31)	Australia	Not reported	Unlimited access to online website, Facebook, smartphone app, group blog, and 3 one-on-one telephone counseling sessions	Interviews (telephone)	14	46
Lim et al. (32)	Australia	Age: 34–35 years % born in Australia: 47–55% Education: 58% University level	2 phases: group delivery (5 group, and 2 telephone sessions) or telephone delivery (7 telephone sessions)	Interviews (telephone and face-to-face group)	Group = 136; Telephone = 29	Group = 48; Telephone = 88
O'Reilly and Laws (33)	Australia	Not reported	Smartphone app (Health eMums) (pilot testing phase—no prescribed intervention dose)	Focus groups	26	40
Walker et al. (34)	US	Age: 31–32 years Ethnicity: 46% White, 21% African-American, 33% Hispanic Education: 8–31% High school graduate or less	Multi-platform (text messaging, website, email, and videos) (observational study on DHI usage-no prescribed intervention dose)	Posted questionnaire and 2 open-ended questions	168	33
Biediger-Friedman et al. (35)	US	Age: 18–47 years Ethnicity: 7% White, 7% African-American, 82% Hispanic Education: 44% University level	Smartphone app (prototype testing phase—no prescribed intervention dose)	Focus groups	61	95
Haste et al. (22)	England	Not reported	Website-delivered consultations with dietitians and exercise experts, videos	Interviews	5	31
Nicholson et al. (36)	US	Age: 32 years Ethnicity: 70% White, 13% African-American, 13% Asian, 17% Hispanic Education: 55% University level	Web-based self-management program combined with text messages and emails	Interviews (face-to-face)	10	44
Vincze et al. (37)	Australia	Age: 32 years Ethnicity: Not reported Education: Not reported	Video coaching (five individual real-time video consultations, consisting of two consultations with an Accredited Practicing Dietitian, two with an Accredited Exercise Physiologist, and one optional self-selected session with either practitioner)	Interviews (telephone)	21	78
Huda et al. (38)	Bangladesh	Age: 15–44 years Ethnicity: Not reported Education: 9.5% completed high school	Mobile platform intervention (bi-weekly voice messaging, fortnightly phone calls, and 3 mobile banking cash transfers)	Interviews (face-to-face), surveys and focus groups	14	4

### Quality Assessment

The quality assessment of included studies is presented in Table 2. All the studies had clear aims, appropriate research designs to address the research aim, appropriate recruitment strategy, clearly reported and justified data collection methods, and had considered the relationship between researchers and participants, with sufficiently rigorous data analysis methods. One study did not clearly meet the criteria for appropriate qualitative methodology due to insufficient details on the process of qualitative data collection (38). One study did not meet the criteria for considering ethical issues as ethics approval was not mentioned (35). Findings were clearly presented in all but three studies (22, 34, 35). The research value was adequately discussed in most studies, although in three studies (33, 35, 38) there was limited discussion on new areas of research identified and how the findings may apply to other populations.

**Table 2:** Quality assessment of included studies.

**Quality assessment criterion**	**van der Pligt et al. (31)**	**Lim et al. (32)**	**O'Reilly and Laws (33)**	**Walker et al. (34)**	**Biediger-Friedman et al. (35)**	**Haste et al. (22)**	**Nicholson et al. (36)**	**Vincze et al. (37)**	**Huda et al. (38)**
Clear aims	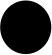	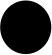	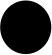	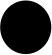	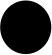	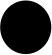	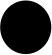	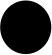	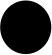
Qualitative	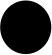	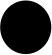	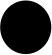	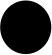	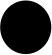	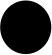	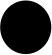	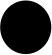	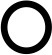
Design	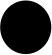	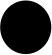	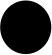	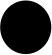	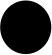	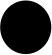	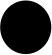	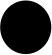	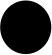
Recruitment	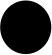	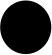	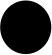	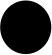	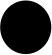	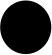	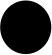	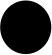	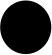
Collection	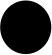	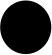	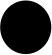	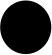	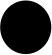	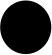	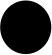	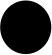	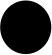
Relationship	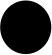	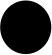	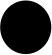	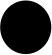	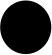	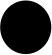	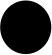	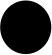	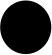
Ethics	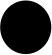	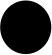	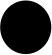	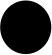	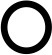	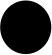	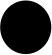	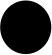	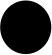
Analysis	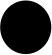	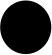	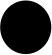	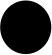	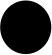	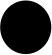	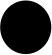	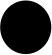	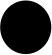
Findings	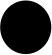	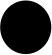	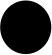	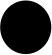	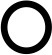	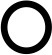	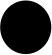	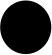	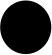
Value	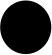	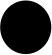	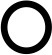	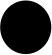	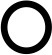	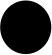	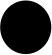	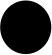	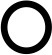

*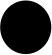 = Met quality assessment criterion 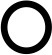 = Unclear or unsure whether quality assessment criterion was met*.

### Perspectives of Postpartum Women on DHIs

Thematic synthesis of the nine included studies that reported the perspectives of postpartum women yielded four themes as summarized in Table 3.

**Table 3:** Summary of key themes, subthemes, and findings on postpartum women's perspective on digital health interventions.

**Theme**	**Subtheme**	**Key findings from individual studies**
Personal facilitators and barriers to lifestyle modification	Facilitators	Resilience and resourcefulness (32) Desire to lose weight, be accountable and learn new knowledge/skills (37) Making it a priority (31)
	Barriers	Childcare, accessibility, confidentiality (32) Lack of time and motivation (31, 35) Poor household planning (35)
Intervention-related strategies for lifestyle modification	Knowledge	Information received was highly important and beneficial (38) Video content was conveniently tailored and comprehensive (37)
	Goal setting	Formal recognition of postpartum phase and emphasis of postpartum goals/objectives (36) Realistic, tailored, and achievable goals were valued (37)
	Health professional support	Personable, encouraging, and knowledgeable dietitian (dietitian video) (37) Fortnightly phone calls were encouraging, supportive, flexible (main enabler), and encouraged accountability (32)
	Feedback and monitoring	Consensus on importance of weight monitoring (31) Tracking of daily weight, exercise, and blood glucose levels (36) Intervention tool allowed health professionals to monitor glucose and weight information (36)
	Prompts and cues	Useful reminders from Facebook (31)
	Peer support	Would use an online forum to communicate with peers (36)
User experience of the technology	Positive	Flexible sessions (37) Ease of navigation, access, quick access, easy to use, well-organized (33, 36, 37) Web-delivered: easy engagement, practical, convenient, fitted into daily routine (22, 34) Online consultation convenient and preferable over face-to-face (37)
	Negative	Sometimes unclear on how to navigate page (33)
Suggestions for improvement	Content	More visual aids (33) More comprehensive information regarding purpose and results of dietary quizzes, BMI explanation, more comprehensive food database, and homemade recipes (33) More personalized lifestyle advice (33)
	Delivery	Ability to print and email (33) Tracking tool for weight and food intake (33) Change website to mobile app for convenience and navigation (22, 31, 36) Use of Facebook as an additional social support tool (33)

Theme 1. Personal Facilitators and Barriers to Lifestyle Modification.

Postpartum women commonly reported personal facilitators and barriers that made it easier or more difficult to engage with lifestyle intervention. These factors appear to be related to their postpartum status specifically, rather than the intervention or its delivery mode. Barriers included lack of time and motivation (35), poor household planning (35), and prioritizing their child's needs over their own well-being, resulting in feelings of worry and guilt when engaging in lifestyle modification (31). For example, household responsibilities were reported as being a cause of missing intervention phone calls (38). On the other hand, numerous facilitators to lifestyle modification were identified. This included personal resources such as resilience (32), support from friends and family (35), peer group support (31, 32), and desire to lose weight or gain knowledge and skills (37).

Theme 2. Intervention-Related Strategies for Lifestyle Modification.

In addition to the personal facilitators and barriers, the included studies reported that postpartum women valued strategies that facilitate behavior change within the interventions they received. Some of these strategies were uniquely supported through electronic means in ways that are not possible otherwise. The behavior change strategies that were valued by postpartum women in DHIs included feedback and monitoring (31, 36); the setting of goals (22, 36, 37); knowledge and information (35, 37, 38); health professional support (31–33, 36, 37); including digital approaches to monitoring of health outcomes such as blood glucose and body weight by health professionals (36); reminders from Facebook (31); and peer support through an online forum (36). There was also an example of a DHI with multiple features contributing to behavior change, such as a mobile application with functions allowing access to exercise information, planning, and tracking, which facilitated the uptake of exercise (35). Women also reported the flexible nature of delivery of DHIs to be highly valued (37). It was unclear why particular behavioral change strategies were preferred, however it appeared to be because they filled an unmet need for the women. However, the importance of the correct intervention dosage was highlighted; in the study by Huda et al. (38), bi-weekly voice messages were perceived as inadequate. The preferred behavior change strategies did not appear to differ across delivery modes.

Theme 3. User Experience of the Technology.

Overall, postpartum women described DHIs as highly acceptable. In general, postpartum women perceived technology as a “natural and comfortable” medium of delivery (22, 33, 36, 37). The interventions were described as easy to use, convenient and practical. There were no qualitative differences in the acceptability by mode, including phone calls, Facebook, mobile applications, web, video, and online peer support. However, different engagement across media according to sociodemographic characteristics was observed (34). In the study by Walker et al. (34), email was preferred by women who reported a higher income, were older and had fewer children, while YouTube was preferred by women with lower education level. This information was derived from a survey of online sources of health information rather than a specific health intervention (34). Despite women being comfortable with using the technology overall, they did report concerns, including technical issues with videos (38) and issues with navigation on a phone application (33).

Theme 4. Suggestions for Improvement.

Postpartum women in the included studies highlighted several ways that future DHIs could be enhanced to facilitate their behavior change in lifestyle interventions. These suggestions centered around personalization or tailoring of interventions (33), and included improvements in the content such as more comprehensive information on the explanation of BMI (33). Suggestions were also provided to improve the delivery of DHIs including the use of Facebook as a social support tool (33), changing from a website to a mobile phone application (22, 31, 36), tracking tools for weight or food intake (33), and risk assessment and screening tools in a diabetes prevention program (33). Flexibility on managing or storing information such as the ability to print and email the information was also recommended (33).

## Discussion

This review aimed to describe the perspectives of postpartum women and health professionals regarding DHIs targeting lifestyle or weight management in the postpartum period. This is the first review on this topic, delivering novel insights into the factors perceived to be most important for postpartum women in DHIs for lifestyle change. However, no studies reporting the perspectives of health professionals were identified in our search. From the studies reporting the perspectives of postpartum women, we identified four themes. These described the personal facilitators and barriers to lifestyle modification, the intervention-related strategies for behavior change, the user experience of the technology, and suggestions for improvement in future DHIs for postpartum weight management.

The barriers noted by postpartum women in studies of DHIs were similar to that described in postpartum lifestyle interventions generally. Poor engagement and high attrition are inherent to lifestyle interventions targeting postpartum women (17, 39). The barriers unique to postpartum women include lack of time due to infant care, low motivation possibly relating to fatigue and sleep deprivation, and changes in priorities due to prioritization of childcare that were identified in the current and previous studies (40, 41). Identifying lifestyle modification as a priority and being resourceful in problem solving may differentiate postpartum women who were engaged from those who were not able to engage (31, 32).

There is evidence that technology may be able to alleviate some of the barriers traditionally reported by postpartum women to engagement with lifestyle management interventions (17). For example, the translation of a group-based diabetes prevention program to telephone-delivered format increased the engagement of postpartum women from 38 to 82% (32). However, due to the qualitative nature of the current analysis, it is unclear if the impact of these ubiquitous postpartum barriers to participants' engagement were quantitatively different in DHIs compared with non-technology-based interventions. Further studies comparing technology vs. in-person interventions in postpartum women are needed to determine whether technology could overcome the barriers faced by this group. DHIs for postpartum women should seek to overcome the barriers of time, motivation, and childcare demands.

Many of the characteristics of the DHIs that were valued by postpartum women included in the studies in our review were related to behavior change strategies (42), for example, setting realistic goals through video consultation with a dietitian (37) and tracking daily weight, exercise, and blood glucose levels in a web-based intervention (36). This is consistent with known key strategies for behavior change, including feedback and goal-setting (43). Other strategies identified in this review, including gaining knowledge and skills (35, 37, 38), being prompted by reminders (31), and getting support from peers (36), have also been found to be important strategies for behavior change in the general population (43). A qualitative study in postpartum women with obesity, but not focused on DHIs, also identified monitoring, gaining knowledge and skills to perform behavior, prompts and cues, and social support to be among the behavior change strategies (44). Many features of DHIs valued by postpartum women in the current review also centered on facilitating the provision of support by health professionals to digital technology users. This was further confirmed in a recent systematic review and meta-analysis which found that support from health professionals was associated with greater weight loss in lifestyle interventions in postpartum women (25). It is apparent, therefore, that technology is merely a delivery medium, and that the core intervention components comprising behavior change strategies remain to be the key ingredients for behavioral outcomes. However, it is important to highlight that technology may provide unique means to facilitate some of these strategies. For example, fortnightly phone calls from a program facilitator provided monitoring and accountability on a flexible schedule (32). Thus, DHIs should utilize the appropriate technology that best facilitates key behavior change strategies for optimal effectiveness.

In our review, women described their experience with technology positively, being easy to engage with, practical, and convenient. This represents postpartum women's experiences over a wide range of DHIs including telephone, mobile phone applications, website, video, social media, and others. A recent systematic review in pregnant women has similarly found DHIs to be acceptable, feasible, and beneficial (45). A qualitative study in pregnant and postpartum women additionally found that all women interviewed unanimously embraced DHIs as a central means to acquire health information and should be included into routine antenatal care procedures in the future (46). In addition, our review revealed a desire by postpartum women for DHIs to capitalize on the functions technology offers in personalizing the intervention. In the future, personalized interventions, that are responsive to individual participant's needs, could be developed using data from the in-built features of smart phones, such as step counters. This approach may overcome a perceived “poor fit” between the digital product and user needs, which is reported as a barrier to intervention engagement in DHIs (24). Co-designing these interventions with input from the women themselves is an important element to ensure personalization and tailoring is achieved and engagement is maximized (26).

There are several strengths in this review. The majority of the included studies were of moderate to good quality, judged by the studies meeting most of the criteria on the CASP tool. The screening was conducted independently by two authors, which minimized bias in assessing eligibility. There was good agreement in the data extraction and appraisal between the authors involved, as well as thematic analyses conducted by two authors. The limitations of this review include the fact that no studies exploring the perspectives of health professionals were identified, which limited our ability to describe this group's perspectives of DHIs. The overall response rate of the included studies were also relatively low at 45%, although this is consistent with other qualitative studies in postpartum women not focusing on DHIs (47, 48). We were also unable to detect clear differences in postpartum women's perspectives attributable to demographic characteristics such as ethnicity or education level as half of the included studies did not report these characteristics (Table 1). No qualitative differences was detected between studies that were mostly represented by White or highly educated participants (32, 36) or studies mostly represented by Hispanic participants or those with low level of education (35, 38) although this remains to be confirmed in further studies. Furthermore, the qualitative review process limited our ability to quantify differences between the different technologies employed in the included studies, as well as to quantify the barriers to engagement with DHIs by postpartum women.

## Conclusions

This systematic review described the perspectives of postpartum women on DHIs targeting lifestyle management in the postpartum period, with no studies reporting on the perspectives of health professionals. Our findings revealed that postpartum women view DHIs as a positive, user friendly, and accepted delivery medium for lifestyle interventions. It was apparent that the barriers reported by women to engaging in postpartum lifestyle interventions are similar to those experienced when participating in non-digital interventions. Therefore, there is a need for future research to identify barriers that can be specifically overcome using DHIs and design interventions appropriately. Furthermore, the behavior change strategies employed in DHIs appear to be consistent with those in non-digital interventions, such as monitoring and feedback, goal setting, inclusion of a credible source (e.g., health professional), and social support. Here, the opportunity to use technology to build on the application of these change techniques within interventions by personalizing the intervention to the user needs must be capitalized on. Consequently, further research is needed to unpack the DHI components that will optimize delivery and engagement in postpartum weight management interventions, with an urgent need to explore the perspectives of health professionals that work with postpartum women. Doing so will contribute to the design of interventions that will promote healthy lifestyles and improve health outcomes for mothers and their children.

## Author Contributions

SL and BH designed the study. AT and SM conducted the screening of titles, abstracts, and full-text articles. AT, SL, and BH extracted the data and appraised the quality of each study. All authors contributed to the drafting of the manuscript and approved of the final version.

### Conflict of Interest

The authors declare that the research was conducted in the absence of any commercial or financial relationships that could be construed as a potential conflict of interest. The handling editor declared a past co-authorship with one of the authors BH.
