# Supplementary material for: Health Professionals' and Postpartum Women's Perspectives on Digital Health Interventions for Lifestyle Management in the Postpartum Period: A Systematic Review of Qualitative Studies
Source: Front Endocrinol (Lausanne). 2019 Nov 8;10:767. doi: 10.3389/fendo.2019.00767 (PMC6856138; doi:10.3389/fendo.2019.00767)
Supplement: Supplementary file 1 [file Table_1.docx]

Supplementary Material

Appendix 1 Search strategy for Medline

|  | **Search terms** |
| --- | --- |
| S1 | MH Postpartum period |
| S2 | TX (postpartum OR post-partum OR postnatal OR post-natal OR puerperium OR postpartal OR post-partal OR lactating OR lactation OR “nursing women” OR breastfeeding OR breast-feeding OR “after birth” OR “following pregnancy” OR postpregnancy OR “post pregnancy” OR “following childbirth” OR “after delivery” OR “post childbirth”) |
| S3 | S1 OR S2 |
| S4 | TX (M-health OR mhealth OR E-health OR ehealth OR ICT OR mobile OR web* OR telephone OR phone* OR digital) |
| S5 | TX (qualitative OR survey* OR interview* OR focus group*) |
| S6 | TX (weight OR “weight retention” OR “weight loss” OR BMI OR “body mass index” OR overweight OR obes* OR “body fat” OR adiposity OR “waist circumference*” OR dietary OR diet OR nutrition OR “healthy eating” OR “physical* activ*” OR exercis*) |
| S7 | S3 AND S4 AND S5 AND S6 |

*Limiters: Date of publication 1990-2019*

Appendix 2 Reasons for exclusion

|  | **Study** | **Reason for exclusion** |
| --- | --- | --- |
| 1 | Beckam J, Green S.L, Nicholson W. Development and a "Hands-On" formative assessment of a web-based pregnancy and postpartum intervention for women with gestational diabetes mellitus. *Diabetes*. 2013;1:A364. | Conference abstract |
| 2 | Cassidy et al. Weight management in Pregnancy: Participants' experiences of 'Healthy Eating and Lifestyle in Pregnancy (HELP)', a maternity care intervention for obese pregnant women. *Pregnancy Hypertension*. 2013;4(3):233-233 | Conference abstract |
| 3 | Cummins J et al. Designing an effective physical activity intervention using the Google TV-putting user input at the forefront. *Journal of Science and Medicine in Sport.* 2012;13(SUPPL.1):S306 | Conference abstract |
| 4 | Goodman A et al. Text4baby as a valuable tool for providers and patients. *Journal of Women’s Health.* 2013;22(3)45-46. | Conference abstract |
| 5 | Guyan A, Bock A, Buback L. Can mobile phones be used to routinely monitor nutrition indicators? Experience from Liberia. *Annals of Nutrition and Metabolism.* 2013;1:1434. | Conference abstract |
| 6 | Koniz-Booher P, Hammink M, Upadhyay A, Beall K. Community-led formative research to determine priority nutrition behaviors for an innovative participatory video feasibility study. *Annals of Nutrition and Metabolism.* 2013;1:1761. | Conference abstract |
| 7 | Maher C, Ziviani J, Miller Y, Olds T, Parkyn H. The internet and postpartum women: Roles, patterns of usage and opportunities for intervention delivery. *Journal of Science and Medicine in Sport*. 2012;15(SUPPL.1):s284-s285 | Conference abstract |
| 8 | Mascarenhas, M. N. Physical activity in motherhood: intervention, trajectory and mixed methods analyses. *Dissertation Abstracts International: The Sciences and Engineering*. 2018;79(4):No pagination. | Conference abstract |
| 9 | McCance D et al. Postnatal lifestyle intervention for overweight women with previous gestational diabetes mellitus (PAIGE): A pilot randomised controlled trial. *Irish Journal of Medical Science.* 2016;185(7):S401. | Conference abstract |
| 10 | Neville C, Irion J, Mallinson T, Abraham K. The effects of a postpartum education program on symptoms and healthcare seeking behaviors in new mothers. *Physiotherapy UK.* 2011;1:eS877-eS878. | Conference abstract |
| 11 | Nicklas J et al. Development and modification of a mobile health program for postpartum women at elevated risk for cardiometabolic disease*. Journal of General Internal Medicine.*2018;33(1):156. | Conference abstract |
| 12 | Nicklas J et al. A web-based lifestyle intervention to decrease postpartum weight retention in women with recent gestational diabetes mellitus: The balance after baby pilot RCT. *Journal of Internal Medicine*. 2013;1:S14 | Conference abstract |
| 13 | Ramos D et al. Choose health la MOMs: A novel approach to postpartum weight loss. *Obstetrics and Gynecology*. 2016;127(Supplement 1):77S-78S | Conference abstract |
| 14 | Van Mulken M. Towards a comprehensive understanding of women’s physical activity behaviour postpartum: Implications for intervention design. *Journal of Science and Medicine in Sport*. 2012;15(Supplement 1):S288. | Conference abstract |

Did not relevant outcome variables (n=3)

| **No.** | **Study** | **Reason for exclusion** |
| --- | --- | --- |
| 1 | Fernandez ID et al. eMoms: Electronically-mediated weight interventions for pregnant and postpartum women. Study design and baseline characteristics. *Contemporary Clinical Trials.* 2015;43:63-74. | Aimed at testing effectiveness against excessive gestational weight gain. Pregnant and postpartum women both recruited. Little qualitative data. |
| 2 | Goode K. Evaluation of a digital health resource providing physiotherapy information for postnatal women in a tertiary public hospital in Australia. *Mhealth*. 2018;4:42. | Unrelated to weight loss and focused on birth recovery instead. |
| 3 | Prieto JT, Zuleta C and Rodriguez JT. Modeling and testing maternal and newborn care mHealth interventions: a pilot impact evaluation and follow-up qualitative study in Guatemala. *Journal of the American Medical Informatics Association*. 2017;24(2):352–360. | Not directly related to postpartum weight loss. Focus was on maternal health and newborn instead. |

Did not report qualitative perspectives on Digital Health Interventions (DHIs) (n=33)

| **No.** | **Study** | **Reason for exclusion** |
| --- | --- | --- |
| 1 | Albright C L et al. Baseline results from Hawaii’s Nā Mikimiki Project: a physical activity intervention tailored to multiethnic postpartum women. *Women and Health.* 2012;52(3):265-291. | Tested DHI efficacy in general and no qualitative perspectives on DHIs |
| 2 | Albright C L et al. Effectiveness of a 12-month randomized clinical trial to increase physical activity in multiethnic postpartum women: results from Hawaii’s Nā Mikimiki Project *Women and Health.* 2014;69:214-223. | Tested DHI efficacy in general and no qualitative perspectives on DHIs |
| 3 | Bensley R J et al. Accessibility and preferred use of online Web applications among WIC participants with Internet access. *Journal of Nutrition Education and Behaviour.* 2014;46(3):S87-S92. | Survey design did not include qualitative perspectives on DHIs |
| 4 | Bishwajit G, Hoque MR, Yaya S. Disparities in the use of mobile phone for seeking childbirth services among women in the urban areas: Bangladesh Urban Health Survey. *BMC Medical Informatics and Decision Making.* 2017;17(1):182. | No qualitative perspectives on DHIs reported |
| 5 | Craigie A et al. Supporting postpartum weight loss in women living in deprived communities: design implications for a randomised control trial. *European Journal of Clinical Nutrition.* 2011;65(8):952-958. | No qualitative data included. |
| 6 | Damron D et al. Factors associated with attendance in a voluntary nutrition education program. *American Journal of Health Promotion.* 1999;13(5):268-275. | No qualitative perspectives on DHIs reported |
| 7 | Downs DS et al. Predictors of Postpartum Exercise According to Prepregnancy Body Mass Index and Gestational Weight Gain. *Journal of Physical Activity and Health*. 2017;14(10):797-807. | No qualitative perspectives on DHIs reported |
| 8 | Guyon A, Bock A, Buback L and Knittel B. Mobile-Based Nutrition and Child Health Monitoring to Inform Program Development: An Experience From Liberia. *Global Health, Science and Practice.* 2016;4(4):661-670. | No qualitative perspectives on DHIs reported. Focused on child health. |
| 9 | Sagedal LR et al. Study protocol: fit for delivery - can a lifestyle intervention in pregnancy result in measurable health benefits for mothers and newborns? A randomized controlled trial. *BMC Public Health*. 2013;13:132 | No qualitative perspectives. Also did not meet participant inclusion criteria as only pregnant women were recruited. |
| 10 | Handley MA et al. Applying the COM-B model to creation of an IT-enabled health coaching and resource linkage program for low-income Latina moms with recent gestational diabetes: the STAR MAMA program. *Implementation Science.* 2016 | No qualitative perspectives on DHIs reported |
| 11 | Kearney MH and Simonelli MC. Intervention fidelity: lessons learned from an unsuccessful pilot study. *Applied Nursing Research.* 2006;19(3):163-166. | No qualitative perspectives on DHIs reported. |
| 12 | Khondabandeh F et al. Effect of educational package on lifestyle of primiparous mothers during postpartum period: a randomized controlled clinical trial. *Health Education Research.* 2017;32(5):399-411. | No perspective/opinions on DHIs collected. |
| 13 | Kim C, Draska M, Hess ML, Wilson EJ and Richardson CR. A web-based pedometer programme in women with a recent history of gestational diabetes. *Journal of the British Diabetic Association.* 2012;29(2):278-283. | Little qualitative opinions reported with primary outcome being change in blood glucose levels. |
| 14 | Kim HK et al. Effects of Online Self-Regulation Activities on Physical Activity Among Pregnant and Early Postpartum Women. *Journal of Health Communication.* 2015;20(10):1115-1124. | No qualitative perspectives on DHIs. Pregnant women also included in study and outcomes not reported separately |
| 15 | Kim J. Survey on the programs of Sanhujori centers in Korea as the traditional postpartum care facilities. *Women and Health*. 2003;38(2):107-17. | No perspective/opinions on DHIs collected. |
| 16 | Lewis BA et al. Examination of a telephone-based exercise intervention for the prevention of postpartum depression: design, methodology, and baseline data from The Healthy Mom study. *Contemporary Clinical Trials.* 2012;33(6):1150-1158. | No perspective/opinions on DHIs collected. |
| 17 | Fjeldsoe, BS, Miller YD and Marshall AL. Social cognitive mediators of the effect of the MobileMums intervention on physical activity. *Health Psychology.* 2013;32(7):729-738 | No perspective/opinions on DHIs collected. Focused on walking/exercise frequency as outcomes instead. |
| 18 | Lewis BA et al. A pilot study evaluating a telephone-based exercise intervention for pregnant and postpartum women. *Journal of Midwifery and Women’s Health.* 2011;56(2):127-131. | No perspective/opinions on DHIs collected. Pregnant women also included in study and outcomes not reported separately |
| 19 | Liu J et al. Preventing Excessive Weight Gain During Pregnancy and Promoting Postpartum Weight Loss: A Pilot Lifestyle Intervention for Overweight and Obese African American Women. *Maternal and Child Health Journal.* 2015;19(4):840-849. | No perspective/opinions on DHIs collected. Pregnant women also included in study and outcomes not reported separately |
| 20 | Logsdon M et al. Preferred Health Resources and Use of Social Media to Obtain Health and Depression Information by Adolescent Mothers. *Journal of Child and Adolescent Psychiatric Nursing.* 2014;27(4):163-168. | No qualitative perspective on DHIs reported. Focus was on depression instead. |
| 21 | Mouratidou T et al. Are the benefits of the 'Healthy Start' food support scheme sustained at three months postpartum? Results from the Sheffield 'before and after' study. *Maternal and Child Nutrition.* 2010;6(4):347-357. | No qualitative perspective on DHIs reported. |
| 22 | Ohlendorf JM. Stages of change in the trajectory of postpartum weight self-management. *Journal of Obstetric, Gynecologic and Neonatal Nursing.* 41(1):57-70. | No qualitative perspective on DHIs reported. |
| 23 | Ohlendorf JM, Weiss ME, Oswald D. Predictors of engagement in postpartum weight self-management behaviours in the first 12 weeks after birth. *Journal of Advanced Nursing.* 2015;71(8):1833-1846. | No qualitative perspective on DHIs reported. |
| 24 | Ohlendorf, JM. Weiss ME, Ryan P. Weight-Management Information Needs of Postpartum Women. *American Journal of Maternal Child Nursing.* 2012;37(1):56-63 | No qualitative perspective on DHIs reported. |
| 25 | Ostbye T et al. Active Mothers Postpartum: a randomized controlled weight-loss intervention trial. *American Journal of Preventative Medicine.* 2009;37(3)L173-180 | No qualitative perspective on DHIs reported. Focused more on community (face-to-face) intervention. |
| 26 | Peterson KE et al. Design of an intervention addressing multiple levels of influence on dietary and activity patterns of low-income, postpartum women. *Health Education Research.* 2002;17(5):531-540. | No qualitative perspective on DHIs reported. |
| 27 | Phelan S et al. Does behavioral intervention in pregnancy reduce postpartum weight retention? Twelve-month outcomes of the Fit for Delivery randomized trial. *American Journal of Clinical Nutrition.* 2014;99(2):302-311. | No qualitative perspective on DHIs reported. Study population also ineligible. |
| 28 | Price S N et al. Content analysis of motivational counseling calls targeting obesity-related behaviors among postpartum women. *Maternal and Child Health Journal.* 2012;16(2):439-447. | No qualitative perspective on DHIs reported. |
| 29 | Reinhardt JA et al. Implementing lifestyle change through phone-based motivational interviewing in rural-based women with previous gestational diabetes mellitus. *Health Promotion Journal of Australia.* 2012;23(1):5-9. | No qualitative perspective on DHIs reported. Focused on reducing gestational diabetes. |
| 30 | Taveras E et al. First Steps for Mommy and Me: A Pilot Intervention to Improve Nutrition and Physical Activity Behaviors of Postpartum Mothers and Their Infants. *Maternal and Child Health Journal*. 2011;15(8):1217-1227. | No qualitative perspective on DHIs reported. Mixture of DHI and face-to-face intervention with separate outcomes not reported. |
| 31 | van der Pligt P et al. A pilot intervention to reduce postpartum weight retention and central adiposity in first-time mothers: results from the mums OnLiNE (Online, Lifestyle, Nutrition & Exercise) study. *Journal of Human Nutrition and Dietetics.* 2018;31(3):314-328 | No qualitative perspective on DHIs reported. |
| 32 | Walker LO, Im EO, Vaughan MW. New mothers' interest in web-based health promotion: association with healthcare barriers, risk status, and user characteristics. *Telemedicine Journal and E-Health. 2012*;18(10):785-790. | No qualitative perspective on DHIs reported directly from postpartum women. |
| 33 | Walker LO, Im EO, Vaughan MW. Communication technologies and maternal interest in health-promotion information about postpartum weight and parenting practices. *Journal of Obstetric, Gynecologic, and Neonatal Nursing: JOGNN*. 2012;41(2):201-215. | No qualitative perspective on DHIs reported directly from postpartum women. Focused on whether or not income level had any correlation with DHI use. |

Did not meet participant inclusion criteria (n=7)

| **No.** | **Study** | **Reason for exclusion** |
| --- | --- | --- |
| 1 | Alexandra Friedman M et al. Reciprocal Peer Support for Post-partum Patients with Diabetes: A Needs Assessment for the Diabetes Buddy Program. *Journal of Community Health.* 2016;41(2):354-358. | Did not meet participant inclusion criteria as pregnant women were recruited. |
| 2. | Biediger-Friedman L et al. A Focus Group Study Observing Maternal Intention to Use a WIC Education App. *American Journal of Health Behaviour*. 2018;42(6):110-123. | Did not meet participant inclusion criteria as not specifically limited to postpartum women but mothers in general. |
| 3 | Bookari K, Yeatman H, Williamson M. Informing Nutrition Care in the Antenatal Period: Pregnant Women's Experiences and Need for Support. *Biomed Research International.* 2017:4856527. | Did not meet participant inclusion criteria as only pregnant women were recruited. |
| 4 | Davis AM et al. Health behavior change in pregnant women: a two-phase study. *Telemedicine Journal and eHealth.* 2014;20(12):1165-1169. | Did not meet participant inclusion criteria as only pregnant women were recruited. |
| 5 | Seward MW et al. Supporting healthful lifestyles during pregnancy: a health coach intervention pilot study. *BMC Pregnancy and Childbirth.* 2018;18(1):375. | Did not meet participant inclusion criteria as only pregnant women were recruited. |
| 6 | Ramachandran A et al. mDiabetes initiative using text messages to improve lifestyle and health-seeking behaviour in India. *BMJ Innovations.* 2018;4(4):155-162. | Did not meet participant inclusion criteria as no postpartum women were recruited. |
| 7 | Halili L et al. Development and pilot evaluation of a pregnancy-specific mobile health tool: a qualitative investigation of SmartMoms Canada. *BMC Medical Informatics and Decision Making.* 2018;18(1):93-95. | Recruited both pregnant and postpartum women but outcomes and analysis were not conducted separately. |

Not related to postpartum weight loss (n=10)

| **No.** | **Study** | **Reason for exclusion** |
| --- | --- | --- |
| 1 | Carr S et al. An evidence synthesis of qualitative and quantitative research on component intervention techniques, effectiveness, cost-effectiveness, equity and acceptability of different versions of health-related lifestyle advisor role in improving health. *Health Technology Assessment.* 2011;15(24):1-284. | Not related to postpartum weight loss |
| 2 | Rhoads, SJ. Exploring implementation of m-Health Monitoring in Postpartum Women with Hypertension. *Telemedicine Journal and eHealth.* 2017;23(10):833-841. | Not related to postpartum weight loss |
| 3 | Soltani H et al. Maternal Obesity Management Using Mobile Technology: A Feasibility Study to Evaluate a Text Messaging Based Complex Intervention during Pregnancy. *Journal of Obesity.* 2015;814830. | Not related to postpartum weight loss |
| 4. | Peragallo U et al. Internet Use and Access Among Pregnant Women via Computer and Mobile Phone: Implications for Delivery of Perinatal Care. *JMIR Mhealth and Uhealth.* 2015;3(1):e25. | Not related to postpartum weight loss and study population restricted to pregnant women. |
| 5 | Ostbye T et al. Internet Use and Access Among Pregnant Women via Computer and Mobile Phone: Implications for Delivery of Perinatal Care. *Military Medicine.* 2003;168(4):320-325. | Not directly related to postpartum weight loss. |
| 6 | Willcox JC et al. Internet Use and Access Among Pregnant Women via Computer and Mobile Phone: Implications for Delivery of Perinatal Care. *JMIR Mhealth and Uhealth* 2015;3(4):e99 | Not related to postpartum weight loss and focus was on antenatal weight management. Only pregnant women were recruited |
| 7 | Hearn L, Miller M and Fletcher A. Online healthy lifestyle support in the perinatal period: what do women want and do they use it? *American Journal of Primary Health.* 2013;19(4):313-318. | Not related to postpartum period but perinatal period instead. |
| 8 | Huberty J, Dinkel D, Beets M and Coleman J. Describing the Use of the Internet for Health, Physical Activity, and Nutrition Information in Pregnant Women. *Maternal and Child Health Journal.* 2013;17(8):1363-1373. | Focused on eating behaviours and physical activities during pregnancy. Pregnant women were only recruited. |
| 9 | Huberty J et al. Development and design of an intervention to improve physical activity in pregnant women using Text4baby. *Translational Behavioural Medicine.* 2016;6(2):285-294. | Focused on improving physical activity levels during pregnancy. Pregnant women were only recruited. |
| 10 | Macleod M et al. Provision of weight management advice for obese women during pregnancy: a survey of current practice and midwives' views on future approaches. *Maternal and Child Nutrition.* 2013;9(4):467-472. | Focused on weight management during pregnancy. |

Not related to DHIs (n=3)

| No | **Study** | **Reason for exclusion** |
| --- | --- | --- |
| 1 | Stringer E, Tierney S, Fox J, Butterfield C, Furber C. Pregnancy, motherhood and eating disorders: a qualitative study describing women's views of maternity care. *Evidence Based Midwifery.* 2010;8(4):112-121. | Not related to DHIs |
| 2 | Price, SN et al. Content analysis of motivational counseling calls targeting obesity-related behaviors among postpartum women. *Maternal and Child Health Journal.* 2012;16(2):439-447. | Not related to DHIs. |
| 3 | Lewis BA et al. Rationale, design, and baseline data for the Healthy Mom II Trial: A randomized trial examining the efficacy of exercise and wellness interventions for the prevention of postpartum depression. *Contemporary Clinical Trials.* 2018;70:15-23. | Not related to DHIs |

Appendix 3. Characteristics of included studies

| First author and year | Setting  (Country) | Study aim(s) | Study Design | Sample Size | Mean age ± SD  (years) | BMI ± SD | Parity | Ethnicity | Mean weeks postpartum or pregnant during recruitment | Digital Health Intervention Platforms | Data Collection  Period | Response Rate |
| --- | --- | --- | --- | --- | --- | --- | --- | --- | --- | --- | --- | --- |
| van der Pligt 2018 | Australia | To describe acceptability and effectiveness of the mum OnLiNE pilot for new mothers | Interviews (telephone) | 14 | NR | NR | Unknown | NR | NR | Unlimited access to online website, Facebook, smartphone app, group blog and 3 one-on-one telephone counselling sessions | NR | 46% |
| Lim 2017 | Australia | To explore the acceptability and engagement of a telephone program for women with previous gestational diabetes | Interviews (telephone and face-to-face group) | Group delivery= 136  Telephone delivery= 29 | Group delivery= 34.1 ± 5.3  Telephone delivery= 34.8 ± 4.8 | Group delivery= 29.2 ± 6.9kg/m2  Telephone delivery= 29.4 ± 6.0kg/m2 | Group delivery= 48% PP & 54% MP  Telephone delivery= 34% PP & 64% MP | Group delivery= 47% born in Australia  Telephone delivery= 55% born in Australia | NR | 2 phases: group delivery and telephone delivery.  Home visits supplemented by telephone calls | Jan 2011-Oct 2015 | Group delivery= 48%  Telephone delivery= 88% |
| O’Reilly 2018 | Australia | To evaluate a pilot smartphone app for women with previous gestational diabetes | Focus groups | 26 | 33.9 | NR | 41% PP & 59% MP | NR | NR | Smartphone app (Health eMums) | Aug-Oct 2015 | 26/65= 40% |
| Walker 2017 | US | To determine new mothers’ preferences for various electronic media and technologies | Posted questionnaire and 2 open-ended questions | 168 but 3 did not use eHealth for health information | White= 31.8 ± 5.5  African American= 31.1 ± 5.5  Hispanic= 31.1 ± 5.3 | White= 43.2% overweight/obese  African American= 60.6% overweight/obese  Hispanic= 56.6% overweight/obese | White= 58.7% PP & 41.4% MP  African American=40% PP & 60% MP  Hispanic= 40% PP & 60% PP | White= 45.5%  African American= 21.2%  Hispanic= 33.3% | White= 9.1 ± 1.9 months  African American= 9.4 ± 2.0 months  Hispanic= 9.3 ± 1.9 months | Multi-platform (text messaging, website, email and videos) | NR | 32.8% |
| Biediger-Friedman 2016 | US  US/Mexico Border | To determine user needs and preferences to inform the first stages of app design and prototyping | Focus groups | 61 | 27.7 | NR | 8% PP & 87% MP | Hispanic= 82%  White=7%  African American= 7%  Other=3% | NR | Smartphone app | 2014 | 61/64= 95.3% |
| Haste 2018 | England | To assess feasibility and acceptability of a web-delivered weight loss intervention for postpartum women | Interviews | 5 | ≥ 18 | BMI ≥ 30 and < 40 | NR | NR | ≥ 3 months but < 2 years | Website and videos | NR | 5/16= 31.3% |
| Nicholson 2016 | US | To assess feasibility, usability and acceptability of a web- based pregnancy and postpartum behavioural intervention | Interviews (face-to-face) | 10 | 33 | NR | NR | African American, White, Hispanic | NR | Web-based combined with text messages and emails | Jan-Apr 2012 | 10/23= 43.5% |
| Vincze 2018 | Australia | To explore postpartum women’s perspectives of engaging with nutritionist and exercise experts through video. | Interviews (telephone) | 21 | 32.3 ± 3.0 | 28.1 ± 3.8 kg/m2 | 1.6 ± 0.9 children or 61.9% PP & 38.1% MP | 90.5% born in Australia  9.5% not born in Australia | 215 ± 61.4 days post-birth | Video coaching | NR | 77.8% |
| Huda 2018 | Bangladesh | To determine the feasibility, acceptability and appropriateness of nutrition intervention using a mobile platform | Interviews (face-to-face), surveys and focus groups | 14 | Range=15-44 | NR | NR | From the Netrokona district, Kendua Upazilla | Currently pregnant or gave birth in the last 6 months | Bi-weekly voice messaging, fortnightly phone calls and 3 mobile banking cash transfers | Feb-Mar 2016 | 14/340= 4.1% |

PP=primiparous; MP=multiparous; BMI = Body Mass Index; NR = Not Reported; SD = Standard Deviation
